# A regression system for estimation of errors introduced by confocal imaging into gene expression data *in situ*

**DOI:** 10.1186/1471-2105-12-320

**Published:** 2011-08-04

**Authors:** Ekaterina Myasnikova, Svetlana Surkova, Grigory Stein, Andrei Pisarev, Maria Samsonova

**Affiliations:** 1 Department of Computational Biology, Center for Advanced Studies, St.Petersburg State Polytechnical University, St.Petersburg, 195251, Russia; 2 Confocal Microscopy and Image Processing Group, Institute of Cytology RAS, St.Petersburg, 194064, Russia

## Abstract

**Background:**

Accuracy of the data extracted from two-dimensional confocal images is limited due to experimental errors that arise in course of confocal scanning. The common way to reduce the noise in images is sequential scanning of the same specimen several times with the subsequent averaging of multiple frames. Attempts to increase the dynamical range of an image by setting too high values of microscope PMT parameters may cause clipping of single frames and introduce errors into the data extracted from the averaged images. For the estimation and correction of this kind of errors a method based on censoring technique (Myasnikova et al., 2009) is used. However, the method requires the availability of all the confocal scans along with the averaged image, which is normally not provided by the standard scanning procedure.

**Results:**

To predict error size in the data extracted from the averaged image we developed a regression system. The system is trained on the learning sample composed of images obtained from three different microscopes at different combinations of PMT parameters, and for each image all the scans are saved. The system demonstrates high prediction accuracy and was applied for correction of errors in the data on segmentation gene expression in *Drosophila *blastoderm stored in the FlyEx database (http://urchin.spbcas.ru/flyex/, http://flyex.uchicago.edu/flyex/). The prediction method is realized as a software tool CorrectPattern freely available at http://urchin.spbcas.ru/asp/2011/emm/.

**Conclusions:**

We created a regression system and software to predict the magnitude of errors in the data obtained from a confocal image based on information about microscope parameters used for the image acquisition. An important advantage of the developed prediction system is the possibility to accurately correct the errors in data obtained from strongly clipped images, thereby allowing to obtain images of the higher dynamical range and thus to extract more detailed quantitative information from them.

## Background

Confocal scanning microscopy is a commonly used method for acquisition of high-quality digital two- and three-dimensional images of molecular biological objects. The high quality of confocal images makes it possible to extract quantitative data at a single cell resolution, the availability of which is a necessary prerequisite for successful systems biology studies. However the data accuracy is limited due to errors that arise in the course of confocal scanning. In our recent papers [1,2] we analyzed the sources of errors introduced by two-dimensional confocal imaging into the data on gene expression *in situ *and described algorithms for estimation and correction of these errors. For example, confocal images are inevitably contaminated by photon shot noise [3] and a common way to reduce the noise is the averaging of multiple separate scans. However, the information about the averaged image will be lost if pixels with high or/and low intensities are clipped in single scans. Image clipping is a form of signal distortion related to the limited grayscale range of an image. Pixel values that exceed an upper threshold of the grayscale range (e.g., 255 for an 8-bit format) are cut-off at the threshold value, all the pixels with negative intensities are set to zero. Such pixels are referred to as *over- *and *under-saturated*, respectively. Averaging of clipped scans results in errors in the data extracted from the averaged image. In our previous work we developed a method [1] based on censoring technique for estimation and correction of this kind of errors, however the method implementation requires not only the averaged image but also all the confocal scans which are not provided by the standard procedure of image acquisition.

The degree of image distortion and hence the size of data error caused by clipping depends on microscope parameters, most of all on the values of gain and offset of the photomultiplier tube (PMT), the detection device to measure photons. These parameters are adjusted to control the dynamical range of an image: the PMT gain (voltage) exponentially amplifies a weak signal, while offset defines the background level of intensities subtracted from the image to increase its brightness. Although the PMT parameters are chosen to ensure that in the averaged image pixels take their value inside the grayscale range and do not look clipped, some of the pixels in single scans may be saturated due to photon noise and clipped off. The adjustment of PMT gain affects signal-to-noise ratio (SNR) in the image amplifying the noise level exponentially. The severity of clipping increases with the increase of gain and offset values, the distortions being the largest when the photomultiplier is adjusted to the limits of its sensitivity. Besides the PMT adjustment SNR can be improved by increase of laser power, however, this approach leads to fluorophore saturation and photobleaching. In practice the laser power is kept at a constant high level and the amount of light admitted to the specimen is reduced through AOTF control, which does not amplify the noise. We have conducted experiments to estimate to what extent other microscope parameters, besides the PMT gain and offset, influence the size of data error.

In the present work we introduce a regression system for prediction of error magnitude in the data extracted from the averaged image. The learning samples are composed of images obtained at different combinations of gain and offset values of three different microscopes. The experiments were designed in a way that for each learning image all the scans were saved as separate image files. The linear regression model involves the values of gain and offset as independent variables while the error value estimated for the given mean intensity level is a dependent variable.

Obviously the magnitude of error may vary among the data obtained with different microscopes and under different experimental conditions, and thus application of the prediction system requires a representative learning sample obtained by the same confocal system and the same scanning experiment as the image subject to error correction. To apply the developed regression system for predicting errors in new data we standardize all our training data obtained with three microscopes; we combine them in one sample and train the system on the combined sample.

The error prediction system was applied to correct errors in the data on expression of segmentation genes in *Drosophila *that are stored in the FlyEx database http://urchin.spbcas.ru/flyex/. This data are widely used in research labs. Our aim was to corroborate the high-precision of the data that was used for construction of the integrated atlas of segmentation gene expression.

The proposed method has important applications. Usually it is recommended to adjust the parameters of microscope to almost avoid pixel saturation in single frames; this approach limits the brightness and contrast of averaged images. The newly developed system provides an opportunity to obtain images in a higher dynamical range and thereby to extract more detailed quantitative information from microscope experiments.

The regression system is implemented as a software tool CorrectPattern freely available at http://urchin.spbcas.ru/asp/2011/emm/.

## Methods

### Algorithm

#### Estimation of between-scan noise

The photon shot noise is an inevitable consequence of the basic properties of confocal microscopy. Among the main advantages of this imaging technology over conventional optical microscopy is the presence of a confocal pinhole, which let only light from the focus plane to reach the detector. Pinhole removes "out of focus" light from the image, thereby decreasing the number of photons reaching detectors. The photon noise arises from a discrete nature and small number of detected photons and in a properly aligned microscope is the major source of errors [3]. This noise is signal dependent and follows the Poisson distribution.

**Figure 1:**
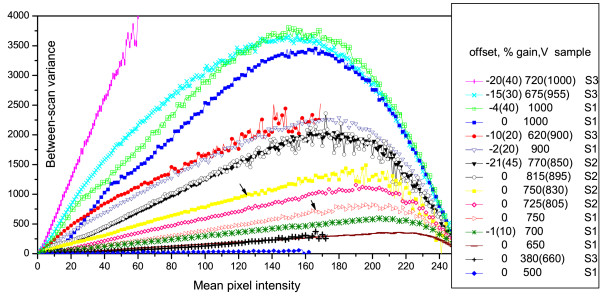
**Photon noise**. Photon noise measured as the between scan variance in selected images from 3 training samples. The curves are arranged in descending order according to the noise level. Offset measured in intensity units is given in parentheses. For images from samples S2 and S3 the rescaled values of gain are given in parentheses, and thus all the gain values are brought to the same scale as in sample S1. The values of noise are computed for all the pixel intensities present in the image. Arrows point to the noise curves computed from images obtained by different microscopes at the same PMT parameters.

The noise level in the averaged image may be characterized by between-scan variance defined for each pixel in the image as a variance of values of the same pixel in all the scans. To illustrate how the between-scan variance depends on the PMT parameters the mean variances are plotted in Figure 1 against the mean pixel values for different combinations of gain and offset. Although the offset adjustment does not directly affect the PMT noise, subtraction of the background from an image decreases mean intensities leaving the noise unchanged and thereby decreasing the signal-to-noise ratio. For example, noise in the image obtained at the gain 1000V and offset -4% is noticeably higher than in the image from the same microscope obtained at the same gain and zero offset. As it is predicted by the optical theory the noise increases exponentially with gain and linearly with offset. It is clearly seen from the figure that in accordance with the properties of the Poisson distribution the variance linearly depends on the mean pixel value at low and intermediate intensities, while at high intensities the variance values dramatically fall as a result of image clipping.

The degree of the averaged image distortion due to clipping is characterized by the fraction of clipped pixels as a function of the pixel intensity. For each pixel the fraction is computed as a number of scans in which this specific pixel is clipped, divided by the total number of scans. Obviously for the pixels with the same intensity in two different images the fraction will be higher for the image with higher noise. The fall of between-scan variances at high intensities is explained by the fact that the fraction of clipped pixels approaches to 1 which results in saturation of the pixel value in the averaged image.

#### Estimation of errors due to image clipping

Quantitative data are read off from the averaged image. The quantification procedure includes the detection of object (nucleus or cell) borders and the subsequent averaging of the values of all the pixels assigned to an object. As a result the data are represented by the mean intensity and coordinates of each object in the image.

The error due to image clipping arises in data extracted from confocal images in the event that these images are obtained by means of averaging the clipped single scans. In the presence of all the scans the error magnitude can be estimated using the method based on the censoring technique [1]. Data errors due to clipping are defined as the absolute difference between the true (unknown) value of the mean intensity and the mean intensity corrupted by clipping that is obtained from the observed averaged image.

To estimate data errors we first introduce *a pixel error *as a value of distortion of the pixel value in the averaged image caused by clipping. Due to clipping at the upper grayscale threshold, *over-saturation*, the pixel intensity is reduced by the value(1)

where *c_a _*is the upper threshold, *f_a_*(*x*) = 1/(2*πσ*) exp [(*x *- *μ*)^2^/*σ*^2^] is the Gaussian distribution density. The parameters *μ *and *σ *are estimated for each pixel by the method of moments as described in [1]. After that the quantities (1) are averaged over all the pixels with equal intensities. Thus for any intensity *k *from the grayscale range [0..*c_a_*] the averaged error, *U_k_*, is defined. We will name this type of error as *upper error*. The error in a data object is given by , where the averaging is performed over all the pixels belonging to the object and *N *is the number of such pixels.

As a result of offset adjustment a certain portion of intensities is subtracted from an image and any pixel value smaller than the subtraction threshold is clipped and set to zero. This type of distortion of single scans, *under-saturation*, yields the overestimated values of pixel intensities in the averaged image. In this case the pixel error is given by(2)

where *c_b _*is threshold defined by the value of offset,  is the Weibull distribution density. The distribution parameters are estimated analogously to those of the Gaussian model (1). The error estimates are averaged over all the image pixels with equal intensities; the averaged pixel error, *lower error*, is denoted as *L_k _*for any *k *∈ [0..*c_a_*]. The data error is also defined in this case as the averaged value of all the pixel errors computed for all the pixels assigned to an object, .

Note, that the magnitude of pixel error is uniquely defined by the level of image noise for a given mean intensity.

Theoretically the method works at any degree of clipping but in practice its application is limited: for example the error estimation is infeasible if the true mean values of a pixel are clipped in all individual scans.

#### Construction of the regression model

The method described in the previous section allows to precisely estimate and correct errors in images and data but its application requires the availability of all the confocal scans. In this section we construct a linear regression model for prediction of magnitude of error in the data extracted from the averaged image based solely on information about microscope parameters. The information about image acquisition is normally contained in scanning protocols saved by the microscope software. Among the microscope parameters the adjustment of PMT gain and offset exerts the greatest influence on the error magnitude and these parameters are incorporated into the regression system as independent variables. As a learning sample we use confocal images scanned at different combinations of gain and offset, for which all the scans are saved along with the averaged images.

The regression algorithm is implemented in several steps. First, for all the elements of the learning sample pixel errors, *U_k _*and *L_k_*, are estimated for all the intensities that are present in the images. Then the regression functions are constructed for each intensity value from the grayscale range. As signal and hence the degree of image distortions depends linearly on offset and exponentially on gain, independent parameters are chosen as the values of offset and exponent of gain. The regression function involves the total estimated distortion caused by under- and over-saturation, *E_k _*= *U_k _*+ *L_k_*, as the dependent variable. For each intensity level, *k *∈ [0..*c_a_*], the linear regression function is defined as(3)

The regression coefficients *β*_offset,*k*_, *β*_gain,*k *_and *β*_0,*k *_are estimated by the least squares method, minimizing(4)

where the summation is done over all the images, elements of the learning sample. Each term includes the values of offset and gain that were applied for the acquisition of the corresponding image.

**Figure 2:**
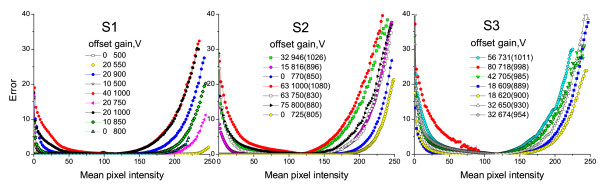
**Estimated error values**. Error values computed by the method (1-2) at different combinations of the PMT parameters for some of the images from 3 training samples. The offset values are measured in intensity units, the PMT voltage (gain) is given in the original and rescaled form (in parentheses). For explanations see text.

Normally a pixel can be noticeably corrupted either by under- or over-saturation and hence only one kind of error, either *U_k _*or *L_k_*, can take considerable values (see Figure 2).

The results of regression estimation are applied to predict the size of errors in data extracted from an averaged image non-belonging to the learning sample, for which single scans are not saved. As a first step, the regression equation (3) is used for prediction of error size in each pixel in the averaged image. Next the predicted values are averaged over all the pixels within the area of each object detected in the image. The errors estimated in this way are used to correct the data distortions that arise due to clipping of single scans both at the highest and the lowest mean intensities.

### Image and data acquisition

Three learning samples were obtained from three confocal microscopes. All the images are two-dimensional of a size 1024 × 1024 pixels and have 8-bit grayscale resolution.

#### S1 sample

The images were obtained by Leica TCS SP5 confocal system (Institute of Cytology RAS, St.Petersburg, Russia). In the scanning experiments we used a specimen prepared from the lily of the valley (Convallaria) root, that is highly autofluorescent in wide spectrum. We also scanned three expression patterns of *Drosophila *embryo, that were also scanned on different microscope system when the S2 sample was constructed. The specimens were scanned 8 times using HCX PL APO 20.0x/0.70 IMM Lbd.BL objective and three lasers (Argon 488 nm, HeNe 543 nm, HeNe 633 nm) with different values of PMT gain and offset listed in Table 1. The power of Argon laser was normally set to 30% of it's maximal value. To check the effect of laser power variation on the noise, one specimen was scanned at 10, 20, 30, 40, 50, and 60 percent laser power at the same values of gain and offset.

**Table 1:** Learning sample S1

Offset\gain,V	500	550	600	650	700	750	800	850	900	1000
0	I(2)	I	I(5)	I	I(2),II(2),III	II	I,II(5),III(3)	II	I,II(3),III	II(3)

-1%(10)	I(2)	I	I	I	I(2),II(2),III	II	I,II(2),III	II	I,II(2),III	-

-2%(20)	I(2)	I	I	I	I(2),II(2),III	II	I,II(3),III	II	I,II(3),III	II

-4%(40)	-	I	-	-	-	-	II	-	II	II

Combinations of PMT gain and offset used for the acquisition of 76 images. The gain values in parentheses are corrected to combine all the samples (see text). Offset values in parentheses are measured in intensity units subtracted from the image. Images are obtained in 3 microscope channels using different lasers (I - 488 nm, II - 543 nm; III - 633 nm). The table entries are the channel numbers in which images at the corresponding combination of PMT parameters are obtained; if more than one image is obtained the number of images is given in parentheses.

#### S2 sample

The experiments were performed at JUC "Chromas" of the St.Petersburg State University, Russia. Eight wild-type (OregonR) *Drosophila melanogaster *blastoderm embryos were immunostained for the expression of *hb*, *gt *and *eve *segmentation genes as described in [4-6]. We used fluorescent labels Alexa Fluor 488 (Invitrogen) for detection of Hb and Cad and Alexa Fluor 555 for detection of Eve and Gt proteins. The embryos were imaged with a HCX PL APO lambda blue 20.0x/0.70 IMM Lbd.BL objective of a Leica TCS SP5 confocal system using Argon 488 and HeNe 543 lasers. Each embryo was stained for the expression of 2 genes, each staining was scanned several times with different values of PMT gain and offset, and for each experiment a series of 8 individual scans was saved together with the averaged image.

In total 59 averaged images (see Table 2) were obtained. To test whether the properties of lasers change with time six stainings were stored and scanned anew with the same values of PMT parameters several months after all the other series of experiments were performed.

**Table 2:** Learning sample S2

offset\gain,V	725-750 (805-830)	770-800 (850-880)	815-850 (895-930)	900 (980)	950 (1030)	1000 (1080)
0	II(2)	II(2)	II(2)	-	-	-

-5%(11)	-	II(4)	II(2)	-	-	-

-10%(21)	II(3)	II(2)	I,II	I	I	I

-15%(38)	II(2)	II(3)	I,II	I(2)	I(2)	I

-20%(50)	II(3)	II(3)	I,II	I	I	I

-25%(63)	II(2)	II(2)	I	I(2),II	I	I

-30%(75)	II	II	-	-	-	-

The sample is composed of 76 images. For notations see caption to Table 1.

#### S3 sample

12 embryos were immunostained for expression of one of four segmentation genes *gt*, *eve*, *hb *and *bcd *applying the same method as described above for construction of S2. Each embryo was scanned several times with different combinations of gain and offset settings (see Table 3). Fluorescent labels used were Alexa Fluor 488 (*bcd *), Alexa Fluor 555 (*eve*, *gt*), and Alexa Fluor 647 (*hb*). Embryo images were taken with the 20X Plan Apo dry objective (numerical aperture 0.7) of a Leica TCS SP2 confocal system at Stony Brook University, NY, USA.

**Table 3:** Learning sample S3

Offset\gain,V	380 (660)	600-640 (980-920)	650-680 (930-960)	700-740 (980-1020)	780-800 (1060-1080)	815-850 (1095-1130)	860-920 (1140-1200)
0-8%(0-16)	I	I(2),II	I(2),II(2)	-	-	-	-

-10-12%(20-24)	-	-	II	II(4)	-	II	-

-25-28%(50-56)	-	-	-	II	II(4)	II(3)	-

-30-35%(60-70)	-	-	-	-	-	II	II(2)

-40-45%(80-90)	-	-	III(2)	III(2)	-	-	-

The sample is composed of 29 images. For notations see caption to Table 1.

The quantitative gene expression levels in nuclei are extracted from the images belonging to S2 and S3 with the use of a nuclear mask as described in [6,7]. The mask is a binary image in which all the pixels located within a nucleus are white and the rest pixels are black. The mask is superposed on the image and the values of pixels belonging to a nucleus are averaged. As a result each nucleus in the expression pattern is characterized by *x *and *y *coordinates and mean intensity level.

## Results

### Training of the system

The regression system is trained on images acquired from three different confocal microscopes. The learning samples S1 and S2 contain images scanned by two different microscopes Leica TCS SP5, the third sample S3 is obtained with a microscope Leica TCS SP2. Details of image acquisition are given in the Methods section. The values of gain and offset used for acquisition of all the samples are presented in Tables 1, 2 and 3. To bring the offset values of different microscopes to the common scale we measure offset in intensity units subtracted from an image. In this way we calculate that 1% offset for microscopes used to acquire S1, S2 and S3 samples corresponds to 10, 2.5 and 2 intensity units, respectively.

First of all, we analyze the photon noise as a function of PMT parameters in all the samples. The noise is estimated as the between-scan variance computed for each element of all the learning samples. The typical behavior of the between-scan variance is shown in Figure 1 and discussed in detail in the Algorithm section. The measured variances are given in Figure 1 for selected values of pixel intensity and PMT parameters that makes it possible to compare the noise level in images obtained with different microscopes and at different conditions. As expected, due to different properties of electronic devices included into the microscope configuration, the noise is not equally defined by the PMT voltage in different microscopes. For example, images obtained at zero offset and equal gain, 750V, from samples S1 and S2 have different level of noise (labeled as errors in the figure). For all our experiments the noise level coincides in images obtained with the same microscope in different channels using different lasers. The power of the laser used for excitation of specimen is another factor that influences the image noise. Although this parameter is normally kept unchanged from experiment to experiment the output laser power may slowly decrease with time as the laser tube ages. We compared the noise in images scanned on different days, even separated by long intervals (up a year), and have established that the noise has not noticeably changed with time. The results of these tests (data not shown) allowed us to assemble all the images obtained by the same microscope into one learning sample. However, the system trained on a learning sample can be only used to predict the error magnitude in data acquired with the same microscope. To be able to predict errors in any data we need to standardize all our training data obtained with three different microscopes and combine them into one sample.

The regression system uses the values of PMT gain and offset as independent variables which means that these parameters uniquely define the predicted error magnitude. To bring the values of these parameters to common scale we represent offset as measured in intensity levels subtracted from an image (see Tables 1, 2 and 3), and further need to find the way how to standardize the values of gain for different PMTs used in different microscopes. As it was already mentioned above, the image noise is unequally defined by the PMT voltage in different microscopes, and even using different lasers in the same microscope, while the noise level completely defines the value of pixel error. Hence it is sufficient to associate the gain values with the level of between-scan noise in images from different learning samples. As noise is known to exponentially increase with increase of gain, to bring the gain values of two samples to correspondence we used additive correction for the gain value in one of the samples. The correction in sample S2 with respect to sample S1 is found to be 80V, such that, for example, the gain value 800V in sample S1 corresponds to 880V in sample S2, which means that these values of gain generate the same level of between-scan noise in images. The correction shift between the gain values in samples S1 and S3 is 280V as the PMT of microscope used to acquire S3 produces much higher noise. For example the level of between-scan noise almost coincide in images from S1 and S3 obtained at zero offset and gain 380V and 650V, respectively (see Figure 1).

Taking into account these corrections we create a common sample consisting of all the learning data obtained from all the microscopes.

### Regression estimation

The combined learning sample is used to fit the regression model (3) introduced in the Methods section. The regression estimation is separately performed for each intensity level *k *∈ [0..*c_a_*]. The value of dependent variables for each element of the learning sample is computed as the sum of upper and lower pixel errors *U_k _*and *L_k_*, if intensity level *k *is present in the image. All the images are 8-bit files, and hence the upper level *c_a _*is equal to 255. However the highest intensities are present just in few images and the upper error values can be estimated for intensities not exceeding ~250. Besides, the highest pixel values are usually very strongly clipped which may lead to unreliable estimates. Examples of error estimates for different combinations of gain and offset are shown in Figure 2.

**Figure 3:**
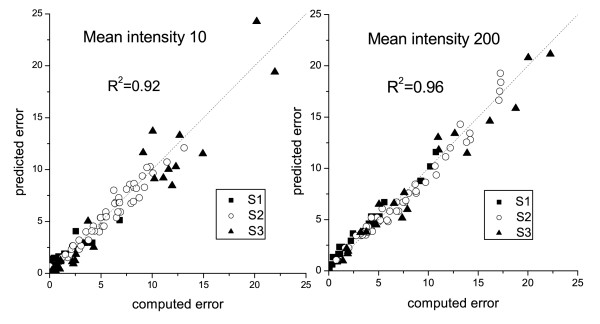
**Results of the regression estimation**. The predicted values of errors are plotted versus the computed ones for three training samples. The results for a low mean pixel intensity are presented in the left panel and for a high mean pixel intensity in the right panel.

The regression coefficients *β*_offset,*k*_, *β*_gain,*k *_and *β*_0,*k *_are estimated by the least square method (4). The results of regression estimation are summarized in Table 4, for the sake of space the estimated values of the coefficients are only given for selected values of intensities *k*. The regression results are visualized for low and high mean intensities in Figure 3. A close to 1 value of the determination coefficient *R*^2 ^is an evidence of adequacy of the regression model and its good prediction properties.

**Table 4:** Results of the regression estimation

intensity	#	*R*^2^	*β*_0_	*β*_offset_	*β*_gain_
0	132	0.941	-0.274	**0.722**	**0.027**

10	132	0.936	**-2.067**	**0.117**	**0.032**

20	132	0.901	**-1.441**	**0.060**	**0.020**

30	132	0.862	**-0.980**	**0.037**	**0.013**

50	132	0.791	**-0.483**	**0.017**	**0.006**

150	98	0.901	**-0.829**	**0.007**	**0.013**

170	98	0.948	**-1.757**	**0.022**	**0.031**

190	94	0.967	**-2.915**	**0.048**	**0.061**

200	89	0.972	**-3.624**	**0.070**	**0.084**

210	89	0.969	**-3.892**	**0.098**	**0.110**

220	85	0.950	**-3.446**	**0.124**	**0.140**

230	73	0.898	**-1.835**	**0.179**	**0.168**

245	37	0.847	**-7.655**	**0.198**	**0.416**

248	12	0.895	-2.569	-0.025	**0.383**

Results of the regression estimation for selected values of the mean pixel intensity. For each mean intensity the number of valid cases, determination coefficient *R*^2^, and estimates of the regression coefficients betas are presented. The significant values of betas are given in bold.

To cross-validate the accuracy of prediction we performed a so-called leave-one-out test. The test uses a single observation from the original sample as validation data and the remaining observations as training data. This procedure is repeated so that each observation in the sample is used once as validation data.

**Figure 4:**
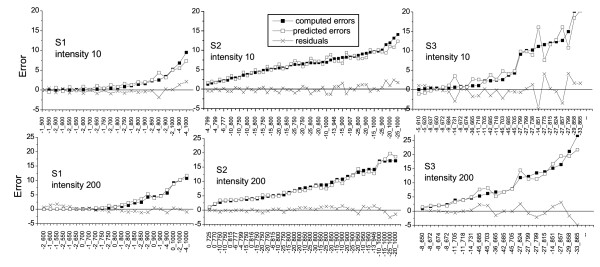
**Results of the cross-validation test**. The computed and predicted values of errors at low and high mean pixel intensities are presented for the images from 3 training samples. If there are several images obtained at the identical PMT parameters in the sample the data are only presented for one of such images. The *x*-axis is labeled by values of PMT offset and gain measured in percents and Volts, respectively.

The test was slightly modified since some images were obtained at the same values of PMT parameters; all such images were together excluded from the training dataset to form the validation sample. At each step of the test procedure we apply the regression system to predict pixel errors for an image from the validation sample. For each pixel value the accuracy of prediction is characterized by the absolute difference between the computed and predicted error values. The cross-validation results are presented in Figure 4. The test confirms high accuracy of error estimation for samples S1 and S2, while for the images from S3 the deviation in error estimation attains 5 units in absolute value. The lower accuracy of error estimation for sample S3 is explained by much higher noise in the images from this sample.

The method was used to predict the sizes of errors in data on expression of 14 segmentation genes in *Drosophila *embryo generated in our previous work and stored in the FlyEx database (http://urchin.spbcas.ru/flyex/; http://flyex.uchicago.edu/flyex/). This dataset consists of about 5000 confocal images, of which 1263 were acquired by microscope and lasers used to generate S3 sample (see the *Methods *section), while the rest images were obtained by microscope and lasers applied to scan S2 sample [4-6]. The images were used to extract quantitative data on segmentation gene expression by the method presented in [6], however this data is corrupted by clipping because all the images were obtained under the standard image acquisition procedure which precludes saving of single scans along with the averaged image.

The data error is usually computed as an average of errors of all the pixels in a data object (in our case an embryo nucleus). However, at high mean intensities this approach is likely to produce unreliable estimates. Due to photon noise the intensities of some pixels in nucleus with high mean intensity may reach values exceeding 250, while estimates of pixel errors are inconsistent at such intensities. In this case it is rather recommended to estimate the data error as the error of a pixel with the intensity equal to the mean intensity of the data object. We have tested this simplified approach on the available data and have observed that the error estimates computed by both methods did not have noticeable differences.

In general, to apply the regression system for prediction of errors caused by clipping in data extracted from a series of 8-bit images scanned by any confocal microscope it is sufficient to bring the values of PMT parameters used for image acquisition to the common scale. For this purpose there is no need to create a full representative learning sample but just to run a specially designed experiment on the same microscope in the same channel and under the same conditions. To measure the value of offset subtracted from images the same staining is scanned twice using the same gain and two different values of offset. The mean difference between the images divided by the difference between the offset values will give the standard measure of offset. To standardize the gain all the confocal scans are saved for an image scanned at zero offset and any given value of gain. Then the between-scan noise is computed as described in and its values are put into correspondence with those computed for our combined sample and presented in Additional file 1, Table S1. The difference between the gain voltage that generates the same level of noise in an image from the combined sample and new experiment will give the correction shift for the gain.

Finally we come to the following scheme of the data error prediction algorithm:

1. Bring the values of PMT parameters used for image acquisition to the standard scale.

2. For any obtained image apply the regression system to to predict pixel errors using the standardized values of gain and offset as input parameters.

3. Compute the sizes of errors caused by clipping by averaging the predicted values over all the pixels within the area of each object detected in the image, or just take the pixel error corresponding to the mean intensity in the object.

### Software tool

The algorithm for prediction of data errors in gene expression patterns is implemented as a software tool CorrectPattern freely available at http://urchin.spbcas.ru/asp/2011/emm/. The main function of the program is to predict and correct errors due to pixel saturation in an input gene expression pattern. Input parameters of the program are the values of gain and offset used for image acquisition. The program provides a tool for automated parameter standardization. A user should provide a series of confocal scans obtained at zero offset for the gain standardization and two averaged images of the same specimen obtained with the same gain and different values of offset for offset standardization. The program computes and saves the corrections for gain and offset that are further used for standardization of the input parameters for any image obtained using the same microscope laser. Output data file is saved in the same format as the input file with the mean intensity values replaced by the corrected ones.

CorrectPattern is realized as a complex of programs in C, Java and JavaScript languages using the three-tier architecture. Program modules are installed on Linux server and use image processing libraries. The user interface is realized in the WEB browser in JavaScript language on the basis of AJAX technology. WEB server is used as an intermediary between the user interface and functional program modules.

### Examples of the method application

#### Correction of gene expression patterns

**Figure 5:**
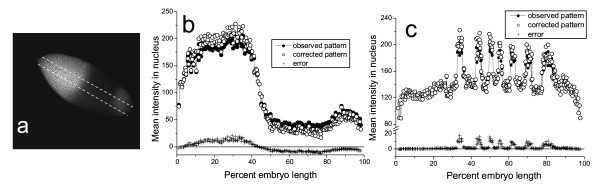
**Correction of errors in quantitative data**. a) Confocal image of *Drosophila *embryo stained for the expression of *hb *gene and scanned with offset -40% and gain 705V. The image belongs to S3. Quantitative data are extracted from the area outlined in the image. b) 1D expression pattern of *hb *gene extracted from the image (black circles) and corrected to eliminate the saturation effect (white circles). Differences between corrected and observed quantitative gene expression data are shown by crosses. c) Quantitative data on *ftz *gene expression extracted from the image taken by the same microscope as sample S2 with offset -2% and gain 1100V (the image is not shown).

The method application is illustrated on two gene expression patterns. An example of error correction in the data extracted from an image belonging to S3 sample is shown in Figure 5b. Predicted error values reached 15-20 units at the highest mean intensities and 12 units at the minimal mean intensities present in the pattern.

An advantage of the proposed method is the opportunity to increase the dynamical range of low signal images and acquire accurate quantitative data from them. The second example illustrates the correction of an expression pattern of fushi tarazu (*ftz*) segmentation gene in *Drosophila *blastoderm obtained using a poor quality antibody. The specimen was scanned by the microscope and laser used to generate S2 sample.

According to the standard procedure for quantitative data acquisition [6] the gain and offset of the microscope photomultiplier should be adjusted so that the maximum level of gene expression corresponds to the maximum level of fluorescence intensity at an 8-bit scale. For immunochemical detection of the gene product we use rat antibody against Ftz [4] and the commercial secondary antibody anti-rat Alexa Fluor 488. Due to the long-term storage, the activity of the primary antibody decreased and even using very high antibody concentrations we had to raise the gain to almost maximum possible value to obtain images of intensity calibrated against the images of FlyEx embryos stained for the *ftz *expression. The noise level in such images is very high that gives rise to high errors in the data. The regression system was applied to correct these errors that reached considerable values of about 17 units at the intensity 200 as shown in Figure 5c.

Another source of errors in the data obtained with the use of the antibody at our disposal is a high non-specific background signal. We applied the method published in [8] to remove the background and normalize the data. Upon the application of this procedure the error magnitudes increased significantly up to 35 units at the intensity 150. This example shows that in cases when there is a need to use high values of PMT parameters the error correction method is very important to make the data suitable for analysis.

#### Estimation of error sizes in the FlyEx dataset

The FlyEx dataset is a valuable source of information about mechanisms of pattern formation in early development. Besides confocal images of gene expression patterns it contains quantitative data extracted from these images, as well as a set of reference images and data representing the most typical expression pattern for a given developmental time. This data attracts attention of many scientific groups, which widely use FlyEx to study the mechanism of pattern formation, infer regulatory interactions in the segmentation genetic network and develop new mathematical models http://urchin.spbcas.ru/flyex/refs.jsp.

In our recent publication [1] we have shown that this data is corrupted by clipping. Notwithstanding the fact that the sizes of the data errors are small due to proper choice of microscope parameters, the errors should be removed as the quality of conclusions drawn critically depends on the data quality. The general-purpose method for correction of pixel saturation requires all the confocal scans to be saved without averaging. The regression system which we have developed allows us to circumvent this limitation, predict the error magnitude in the data extracted from the average image and apply the error correction procedure to the whole dataset.

The important corollary of extension of the method to predict the error sizes on the whole dataset is the opportunity to accurately estimate the sizes of these errors. Almost all the images from the FlyEx dataset were acquired with the gain values within the range from 450V to 680V and offset values under 45%. The predicted error values do not exceed 7-8% of the highest mean intensity present in expression pattern. The lowest value of mean intensity in nucleus is never zero due to inevitable presence of non-specific background staining [8] in the embryo. The background level is determined by the quality of antibodies used for staining, and in our data it varies between 2 and 100 units. The predicted errors take values not higher than 2-3 units in expression patterns with low background and are negligibly small in the case of high background. The detailed results of the error estimation are given in Additional file 2, Figure S1; Additional file 3, Figure S2 and Additional file 4, Figure S3. Errors at low intensities may slightly affect the estimation of background level but are small enough not to noticeably corrupt the pattern after background subtraction.

## Discussion

In our recent publication [1] we described a new method for estimation and correction of errors in the quantitative data extracted from clipped confocal images. The method was applied to the data on segmentation gene expression in *Drosophila*. A necessary requirement for the method application is availability of all the individual scans that usually are not saved but directly averaged by the microscope software to reduce the photon noise in images. Due to this requirement the method could not be used to correct errors in data obtained by the standard scanning procedure; the method only allows to determine the range of settings that provides acceptable level of errors in a specific microscope.

To extend the applicability of the method we have created a linear regression system and software to predict the magnitude of errors in the data obtained from a confocal image based on information about microscope parameters used for the image acquisition. The system was trained on three samples of images obtained from different microscopes with different combinations of the PMT gain and offset adjustments. As adjustment of PMT gain and offset to the same values in different microscopes produces different level of noise, the scales of these parameter were calibrated to achieve standardization. The standardized parameter values were used by the regression system as independent variables.

To estimate regression function each image in a training sample was saved together with all the individual scans. The computed errors were included as dependent variables into the regression model taking into account a known fact [3] that the error size depends linearly on the offset value and exponentially on the gain value. The system predicts the magnitude of errors in data extracted from an 8-bit image obtained by a confocal microscope using the values of standardized PMT parameters as input. The cross-validation tests demonstrated high accuracy of predictions.

It should be stressed that the standardization of the microscope parameters is very important as it puts into correspondence the properties of images obtained with different microscopes, lasers and under different experimental conditions. All what is needed is to perform a simple experiment to measure the photon noise via the estimation of between-scan variance and standardize the parameter values used for image acquisition against the scale utilized in the training sample. Upon that the regression system can be used on the data extracted from a series of images obtained at the same conditions.

We envisage one additional application of the regression system developed in this work. This system allows a user to extract more detailed quantitative information from the images, thereby increasing the accuracy of gene expression data. The confocal scanning experiment directed to the acquisition of quantitative gene expression data possesses certain specific features. For example, images used to acquire data on segmentation gene expression in *Drosophila *embryo are standardized against the image of an embryo exhibiting the pattern characteristic of maximal expression, that is normally observed at the late stages of development of a wild type embryo. The gain and offset values of the microscope photomultiplier are adjusted for this embryo and kept constant in all the series of scanning experiments. Because of this arrangement it may happen that images of embryos at early stages of development, and especially of mutants, are of very low contrast since the level of gene expression in these embryos is low. This is especially typical for images of expression patterns in embryos stained with the antibodies of poor quality, that give rise to a high nonspecific background. To be able to extract more detailed information from such images it is necessary to increase their intensity range by setting high values of PMT parameters; however this may lead to pixel saturation and errors in quantitative data extracted from the images. Our regression system provides means to estimate and correct errors in data obtained with an extended range of microscope parameters and hereby makes it possible to obtain more accurate quantitative information on gene expression.

## Conclusions

• A regression system is created for error magnitude prediction in data obtained from an 8-bit confocal image. The prediction is based on information about microscope parameters used for image acquisition.

• The method demonstrates high prediction accuracy and was applied for correction of errors in the data on segmentation gene expression in *Drosophila *blastoderm stored in the FlyEx database (http://urchin.spbcas.ru/flyex/, http://flyex.uchicago.edu/flyex/).

• An important advantage of the developed prediction system is the possibility of error correction in data obtained from strongly clipped images, thereby permitting acquisition of higher dynamic range images, which would aid extraction of more detailed quantitative information.

• The system is realized as a software tool CorrectPattern freely available at http://urchin.spbcas.ru/asp/2011/emm/.

## Competing interests

The authors declare that they have no competing interests.

## Authors' contributions

All the authors participated in the design of the study and wrote the manuscript together. All authors read and approved the final manuscript.

## Supplementary Material

Additional file 1 **Photon noise**. Logarithm of the noise level computed at different values of mean pixel intensity in images obtained at zero offset and standardized values of gain. Noise values are only given for pixel intensities almost non-corrupted by pixel saturation Click here for file

Additional file 2 **Predicted errors in the FlyEx data (channel 1)**. Error magnitudes predicted for data on expression of segmentation genes in *Drosophila *stored in the FlyEx database. The embryos were imaged with Leica TCS SP2 confocal system at Stony Brook University using Argon 488 nm laser. The error values are computed at mean intensity levels 10 and 200. In gene expression patterns that due to high non-specific background do not contain nuclei with the mean intensity equal to 10 the error magnitudes are not shown. Click here for file

Additional file 3 **Predicted errors in the FlyEx data (channel 2)**. Error magnitudes predicted for data on expression of segmentation genes in *Drosophila *stored in the FlyEx database. The embryos were imaged with Leica TCS SP2 confocal system at Stony Brook University using HeNe 543 nm laser. For notations see Additional file 2, Figure S1. Click here for file

Additional file 4 **Predicted errors in the FlyEx data (channel 3)**. Error magnitudes predicted for data on expression of segmentation genes in *Drosophila *stored in the FlyEx database. The embryos were imaged with Leica TCS SP2 confocal system at Stony Brook University using HeNe 633 nm laser. For notations see Additional file 2, Figure S1. Click here for file
