# Supplementary material for: A regression system for estimation of errors introduced by confocal imaging into gene expression data in situ
Source: BMC Bioinformatics. 2011 Aug 4;12:320. doi: 10.1186/1471-2105-12-320 (PMC3169536; doi:10.1186/1471-2105-12-320)
Supplement: Additional file 1 — Photon noise. Logarithm of the noise level computed at different values of mean pixel intensity in images obtained at zero offset and standardized values of gain. Noise values are only given for pixel intensities almost non-corrupted by pixel saturation [file 1471-2105-12-320-S1.PDF]

gain,V\intensity	60	80	100	120	140	150
500	2.7	3.1	3.2	3.6	3.8	3.9
550	3.3	3.7	3.9	4.1	4.3	4.4
600	3.8	4.1	4.3	4.5	4.7	4.8
650	4.5	4.8	5.1	5.3	5.4	5.5
700	4.9	5.2	5.5	5.6	5.8	5.9
750	5.4	5.7	5.9	6.2	6.3	6.4
800	5.9	6.2	6.4	6.6	6.8	6.9
850	6.3	6.6	6.8	7.0	7.1	7.2
900	6.6	7.0	7.2	7.4	7.5	7.6
1000	7.3	7.6	7.8	8.0	8.1	8.2
1050	7.6	7.8	8.0	8.1	8.2	8.3
1100	7.8	8.0	8.2	8.4	8.5	8.6
1200	8.1	8.2	8.4	8.6	8.7	8.8

Table S1. Logarithm of the noise level computed at different values of mean pixel intensity in images obtained at zero offset and standardized values of gain. Noise values are only given for pixel intensities almost non-corrupted by pixel saturation.
